# Obesity definition by percentage of body fat compared to body mass Index in a kidney transplant transition clinic

**DOI:** 10.3389/fped.2026.1705330

**Published:** 2026-09-09

**Authors:** P. Chaudhary, D. Pemmasani, A. S. Friederich, C. Nailescu

**Affiliations:** Riley Hospital for Children, Department of Pediatrics, Indiana University, Indianapolis, IN, United States

**Keywords:** adolescent and young adult (AYA), kidney, metabolic syndrome, obesity, transplant

## Abstract

**Purpose:**

Obesity is defined by body mass index (BMI), a poor marker of adiposity that does not take into account sex differences. It was recently shown in a large database of adults that percentage of body fat (%BF) can also be used to define clinically relevant overweight/obese status with sex-specific cut-offs. This definition was never tested in adolescent and young adult (AYA) kidney transplant (KT) recipients.

**Methods:**

We compared the %BF method of defining obesity with the BMI-based method in a population of AYAs belonging to a KT transition clinic. The %BF was measured using a total body composition scale (Body Composition Analyzer, Tanita Corporation, Tokyo) based on the bioelectrical impedance analysis.

**Results:**

Thirty-five patients (18 females, 17 males; mean age 19.5 ± 2.5 years) were enrolled. Hypertension was present in 54.3%; metabolic syndrome in 25.7%. BMI classified 42.9% [95% CI: 28.0–59.1%] as overweight/obese vs. 28.6% [95% CI: 16.3–45.1%] by %BF (McNemar *p* = 0.125). %BF was significantly higher in females (30.1 ± 6.1%) than males (23.3 ± 7.9%; *p* < 0.01). BMI–%BF correlation was stronger in males (*r* = 0.903) than females (*r* = 0.777).

**Conclusions:**

Overweight and obesity were common in KT AYA recipients. Cardiovascular risks are common. The BMI-based method appeared to possibly overestimate obesity, although statistical significance was not attained, likely due to the small sample size. Further studies are needed to confirm if %BF is more accurate in determining obesity compared to BMI in this population.

## 1 Introduction

Obesity is an increasing global health problem leading to cardiovascular, metabolic, mental health and social complications (1). Unfortunately, obesity is also becoming a worrisome pediatric problem as it is estimated that soon, by 2030, there will be 254 million children and adolescents with obesity worldwide (2). Unfortunately, obesity is also prevalent in pediatric kidney transplant (KT) candidates, as well as recipients.

In pediatric KT candidates, obesity seems to be very common, with studies citing prevalence of 12%–15% in the chronic kidney disease and end-stage renal disease populations (3, 4). This is a problem, because obesity poses a risk for multiple complications when it comes to KT, which is ultimately the final treatment plan for such patients. Short-term post-KT complications primarily include delayed-graft function, impaired wound healing and infections, while long-term complications include cardiovascular disease and shorter graft survival (5–7). In fact, many pediatric KT centers consider obesity as a contraindication to transplantation, generally having a center-specific cut-off limit mostly based on Body Mass Index (BMI) (8). Thus, pediatric patients suffering from obesity face barriers getting to KT and often remain on dialysis for a long time, which negatively impacts their life expectancy and quality of life in a profound way (9).

In pediatric KT recipients, obesity prevalence seems to almost double, reported to be 17% at 6 months and 29% at 1 year following transplant, being worse in centers that use steroids as part of the maintenance immunosuppression (10). Obesity in pediatric KT recipients is associated with hypertension, insulin resistance, hyperlipidemia, atherosclerosis, proteinuria and graft failure (5, 7). In addition, patients with obesity and actually with full metabolic syndrome have a higher rate of deceased donor grafts, acute rejection episodes, and receive more methylprednisolone pulses; such patients also have a significantly higher pretransplant BMI than patients without MS (11). It was recently described that approximately one-fifth of patients with pediatric kidney transplants have the full metabolic syndrome (12).

The current criteria generally used to categorize obesity in children and adolescents are based on the American Academy of Pediatrics definition and are based on BMI. Overweight is characterized by a BMI between 85th percentile to less than 95th percentile for age (25–29.9 Kg/m^2^). Obesity Class 1 is characterized a BMI between 95th percentile to less than 120% of the 95th percentile for age (30–34.9 Kg/m^2^). Obesity class 2 is characterized by a BMI between 120% to less than 140% of the 95th percentile for age or BMI ≥35 to <40 Kg/m^2^. Obesity Class 3 is characterized by a BMI equal to 140% of the 95th percentile for age or over 40 Kg/m^2^ (13). It is to be noted that this definition of obesity does not take into account at all any sex-specific differences between females and males.

However, BMI might not be ideal to estimate obesity. First, BMI does not take muscle mass and bone mass into consideration (14). Secondly, BMI does not take into account the differences in adiposity between males and females (higher adiposity in females vs. higher muscle mass in males) (15). It has been proposed that waist circumference might be a more accurate measure of obesity, as it is directly associated with increased cardiovascular risk (16). Recently, percentage of body fat (%BF) has been studied and deemed to possibly be a more accurate method to define obesity in adults (17). There are multiple ways to measure %BF, including bioelectrical impedance analysis, dual-energy x-ray absorptiometry and hydrostatic weighing (18, 19). The problem is that such methods are generally not very practical, certainly requiring more equipment which can be expensive and are more time-consuming than measuring BMI, which simply requires a weight and a height measurement.

Currently there is no consensus among national organizations on definition of obesity using %BF. However, a very recent study from National Health and Nutrition Examination Survey (NHANES) conducted between 1999 and 2018 in 16,918 adults (8,734 men and 8,184 women), ages 18–85 years, proposed a new definition for obesity based on %BF in adults (17). According to this study, clinically relevant “overweight” can be defined as 25% and 36% BF for men and women, respectively, and “obesity” as 30% and 42% BF for men and women (17). This newer definition of overweight/obesity which is sex-specific has never been characterized or tested in adolescent and young adult (AYA) KT recipients.

Our aim was to test how feasible measuring %BF can be in a clinical setting such as our AYA KT Transition Clinic using a very simple and easy to use body composition scale. We hypothesized that the newer method based on %BF will include less patients in the overweight/obesity categories compared to the traditional BMI-based method in this population. In addition, it will be more useful to provide clinically relevant sex-specific guidance about obesity categorization.

## 2 Methods

A single-center, cross-sectional observational study was conducted from May 2024 to September 2025 after Indiana University Institutional Review Board approved the study. All AYAs who had undergone a KT and were seen in the Kidney Transition Clinic at our institution in that time frame were approached and consented. We decided to focus on this age- group as the AYA population is very unique and is not well-studied. Our Transition Clinic with our multi- disciplinary team, not only provides them with the needed medical management but also focuses on their well-rounded care, covering aspects like reproductive health, behavioral and psychosocial well-being, that affects their nutrition, medication adherence and other transitional vulnerabilities.

We measured their total body fat using bio-impedance analysis with a total body composition scale (Body Composition Analyzer, Tanita MC-980 plus, Tokyo, Japan). Practically speaking, the subject stood barefoot on foot pads (electrodes through which the scale sends a small, harmless electrical current through the body). At the same time, the subject held on to electrode handles (which transmit the electrical current back to the scale). All measurements were performed by the same team member at approximately the same time of the day (8am, after patients had their blood drawn pre-clinic visit). The scale underwent once a month quality checks. We categorized patients into underweight, normal weight, overweight and obese according to the definition proposed by Potter et al. (17).

In addition, we measured weight and height to calculate their BMI and based on that we categorized them into underweight, normal weight, overweight and obese according to the definitions for pediatrics up to age 18 and for adults for the patients over 18 (20, 21). Finally, we collected data to help identify the presence of metabolic syndrome according to pediatric definitions for patients up to age 18 and adult population definitions for patients 18 and above (20, 21). There were two deviations from the well-described definition of metabolic syndrome. First, we used Hemoglobin A1c (HbA1c) as a substitute for fasting glucose due to the impracticality of obtaining fasting glucose in our clinic. Secondly, we did not obtain measurements of abdominal adiposity, as our subjects in general refused this part of the evaluation. In addition, the lipid levels were not obtained while fasting. Hypertension was defined as having a BP over the 90th percentile for age, sex and height or over 130/80 mmHg, whichever is lower, or on anti-hypertensive medication. Elevated triglycerides were considered over 130 mg/dL for patients less than 18 and over 150 for patients over age 18, or taking medication for it. Low high-density lipoprotein (HDL) was considered less than 40 mg/dL for patients less than 18; for patients over age 18, abnormal was considered less than 40 mg/dL in males and less than 50 mg/dL in females, or taking medication for it. Elevated HgbA1c was considered ≥5.7%). We compared the differences in the two variables, %BF and BMI, when categorized under normal, overweight, and obese categories based on the current pediatric and adult definitions as appropriate for both variables (17, 20, 21). We also examined differences based on sex.

Patients were all on a similar immunosuppressive regimen in adherence to the current practices of our program that relies on a combination use of a calcineurin inhibitor (Tacrolimus) and an anti-metabolite (Mycophenolate Mofetil). Our current standard of practice does not use corticosteroids for long term management of transplant patients.

For statistical analysis, agreement between methods was assessed by McNemar's exact test and weighted Cohen's kappa (*κ*, quadratic weights, 95% bootstrap CI). Pearson correlations were computed with Fisher *z*-transformation CIs; a BMI×Sex interaction *F*-test assessed whether the BMI–%BF slope differed between sexes. Bland–Altman analysis (*z*-standardized values) examined systematic bias. *Post-hoc* power was estimated by Monte Carlo simulation (50,000 replications). Normality was assessed by Shapiro–Wilk test; %BF was normally distributed (*W* = 0.971, *p* = 0.469). Normally distributed variables are reported as mean ± SD and compared by *t*-test; non-normally distributed variables are reported as median [IQR] and compared by Mann–Whitney *U*. Missing data were handled by complete-case analysis; primary outcomes (BMI, %BF) were complete for all 35 participants, with secondary variables missing in ≤8.6% of cases.

## 3 Results

Of 40 approached patients, 35 consented (17 males, 18 females; mean age 19.5 ± 2.5 years; range 15–25) (Table 1) The reasons for refusal were not completely understood, but they seen to have been related to weight-sensitivity issues. %BF measurement was completed during the routine clinic visit, averaging <5 min. Median time post-transplant was 10.4 ± 4.9 years (Table 1). Hypertension was present in 54.3%; abnormal HbA1c in 14.7%, low HDL in 48.5%, and elevated triglycerides in 54.5%. Metabolic Syndrome was present in 25.7% (Table 1).

**Table 1:** Demographics.

Characteristic	Value
Total enrolled	35
Age, mean ± SD (range)	19.5 ± 2.5 years
Sex (%): Male/Female	17 (48.6%)/18 (51.4%)
Age group: Adolescent (<18)	9 (25.7%)
Age group: Young Adult (18–25)	26 (74.3%)
Race: White Non-Hispanic/ White Hispanic/ Black Non-Hispanic/Other	22 (62.9%), 7 (20.0%), 5 (14.3%), 1 (2.9%)
Height, cm (mean ± SD)	162.8 ± 10.1
Weight, kg (mean ± SD)	73.7 ± 26.4
BMI, kg/m^2^ (mean ± SD)	25.5 ± 5.1
BMI-SDS (BMI Standard Deviation Score) (mean ± SD)	0.7 ± 1.1
Height-SDS (mean ± SD)	−1.1 ± 1.3
Growth impaired (ht z < −2)	3 patients (17.6% of ≤20 yrs)
% Body Fat (mean ± SD)	26.8 ± 7.7
Fat Mass, kg (mean ± SD)	20.2 ± 9.8
BMI: Normal/Overweight/Obese	20 (57.1%)/8 (22.9%)/7 (20.0%)
BMI Overweight + Obese	15/35 (42.9%)
%BF: Normal/Overweight/Obese	25 (71.4%)/6 (17.1%)/4 (11.4%)
%BF Overweight + Obese	10/35 (28.6%)
S. Creatinine, mg/dL (mean ± SD)	1.6 ± 1.0
eGFR, mL/min/1.73m^2^ (mean ± SD) (CKiD U25 formula)	64.5 ± 23.4
CKD G1–G2 (eGFR ≥60)	22 (62.9%)
CKD G3 (eGFR 30–59)	8 (22.9%)
CKD G4–G5 (eGFR < 30)	4 (11.4%)
HbA1c elevated (≥5.7%)	5/34 (14.7%)
HDL low (sex-specific cutoffs)	16/33 (48.5%)
Triglycerides ≥150 mg/dL	18/33 (54.5%)
Hypertension	19/35 (54.3%)
Metabolic Syndrome (YES)	9/35 (25.7%)
Years post-transplant (mean ± SD)	10.4 ± 4.9
Graft type: Deceased/Living	21 (60.0%)/13 (37.1%)
Primary disease: Renal Dysplasia	15/35 (42.9%)
Primary disease: Cortical Necrosis	3 (8.6%)
Primary disease: Focal Segmental Glomerulosclerosis	2 (5.7%)
Primary disease: Obstructive Uropathy	2 (5.7%)
Primary disease: Other/rare	13 (37.1%)
Rejection episodes (mean ± SD)	1.2 ± 1.0
≥1 rejection episode	24/34 (70.6%)

%BF showed significant sex differences: males 23.3 ± 7.9%; females 30.1 ± 6.1% (*p* < 0.05) (Table 2).

**Table 2:** Descriptive statistics by sex.

Variable	Males (*n* = 17)	Females (*n* = 18)	*p*-value	Test
Age (years)	20.0 ± 2.6	19.1 ± 2.5	0.309	*t*-test
Height (cm)	166.4 ± 9.2 (*n* = 12)	159.3 ± 10.1 (*n* = 12)	**0** **.** **038** *	M–W
Weight (kg)	81.8 ± 27.2	66.0 ± 23.8	**0** **.** **010** *	M–W
BMI (kg/m^2^)	26.5 ± 5.5	24.6 ± 4.7	0.292	*t*-test
% Body Fat	23.3 ± 7.9	30.1 ± 6.1	**0** **.** **008** **	*t*-test
Fat-Free Mass (kg)	61.9 ± 19.3	45.8 ± 16.4	**<0** **.** **001** ***	M–W
Muscle Mass (kg)	60.8 ± 20.4 (*n* = 13)	46.3 ± 17.6 (*n* = 13)	**0** **.** **004** **	M–W
S. Creatinine (mg/dL)	1.7 ± 1.1 (*n* = 17)	1.6 ± 0.9 (*n* = 17)	0.605	M–W
eGFR (mL/min/1.73m^2^) (CKiD U25 formula)	69.2 ± 23.4 (*n* = 17)	59.9 ± 23.2 (*n* = 17)	0.133	M–W
Years post-transplant	9.9 ± 4.7	10.8 ± 5.2	0.582	*t*-test
HbA1c (%)	5.2 ± 0.4 (*n* = 17)	5.7 ± 1.4 (*n* = 17)	0.297	M–W
Triglycerides (mg/dL)	169.3 ± 77.2 (*n* = 17)	200.3 ± 180.3 (*n* = 16)	0.943	M–W
BMI-SDS (≤20 yrs)	0.7 ± 1.3 (*n* = 11)	0.6 ± 0.9 (*n* = 13)	0.837	*t*-test
Height-SDS (≤20 yrs)	−1.2 ± 1.5 (*n* = 8)	−1.1 ± 1.1 (*n* = 9)	0.743	M–W
BMI OW/Obese	10/17 (58.8%)	5/18 (27.8%)	0.130	*χ* ^2^
%BF OW/Obese	7/17 (41.2%)	3/18 (16.7%)	0.219	*χ* ^2^
Metabolic Syndrome YES	5/17 (29.4%)	4/18 (22.2%)	0.921	Fisher
Hypertension	10/17 (58.8%)	9/18 (50.0%)	0.854	Fisher

Values are mean ± SD unless noted. M–W = Mann–Whitney *U* test. %BF significantly higher in females (expected physiological difference). Muscle mass, Fat-free Mass, and weight significantly higher in males.

Bold values indicate statistical significance based on the respective *p*-values.

* *p* < 0.05.

** *p* < 0.01.

*** *p* < 0.001.

**Figure 1:**
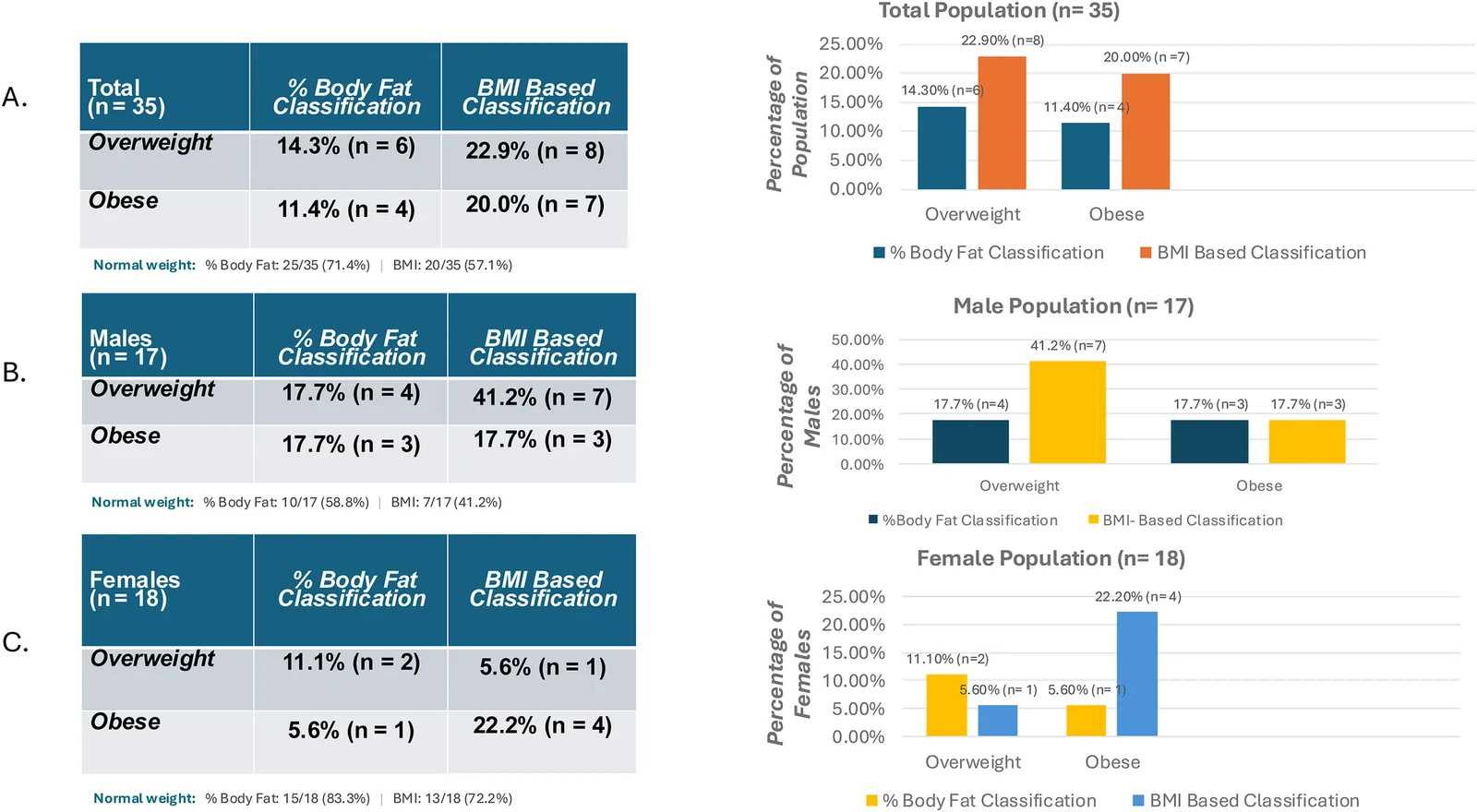
Percentage of patients considered overweight or obese based on % BF method vs. BMI Method: **(A)** overall; **(B)** males; **(C)** females.

**Figure 2:**
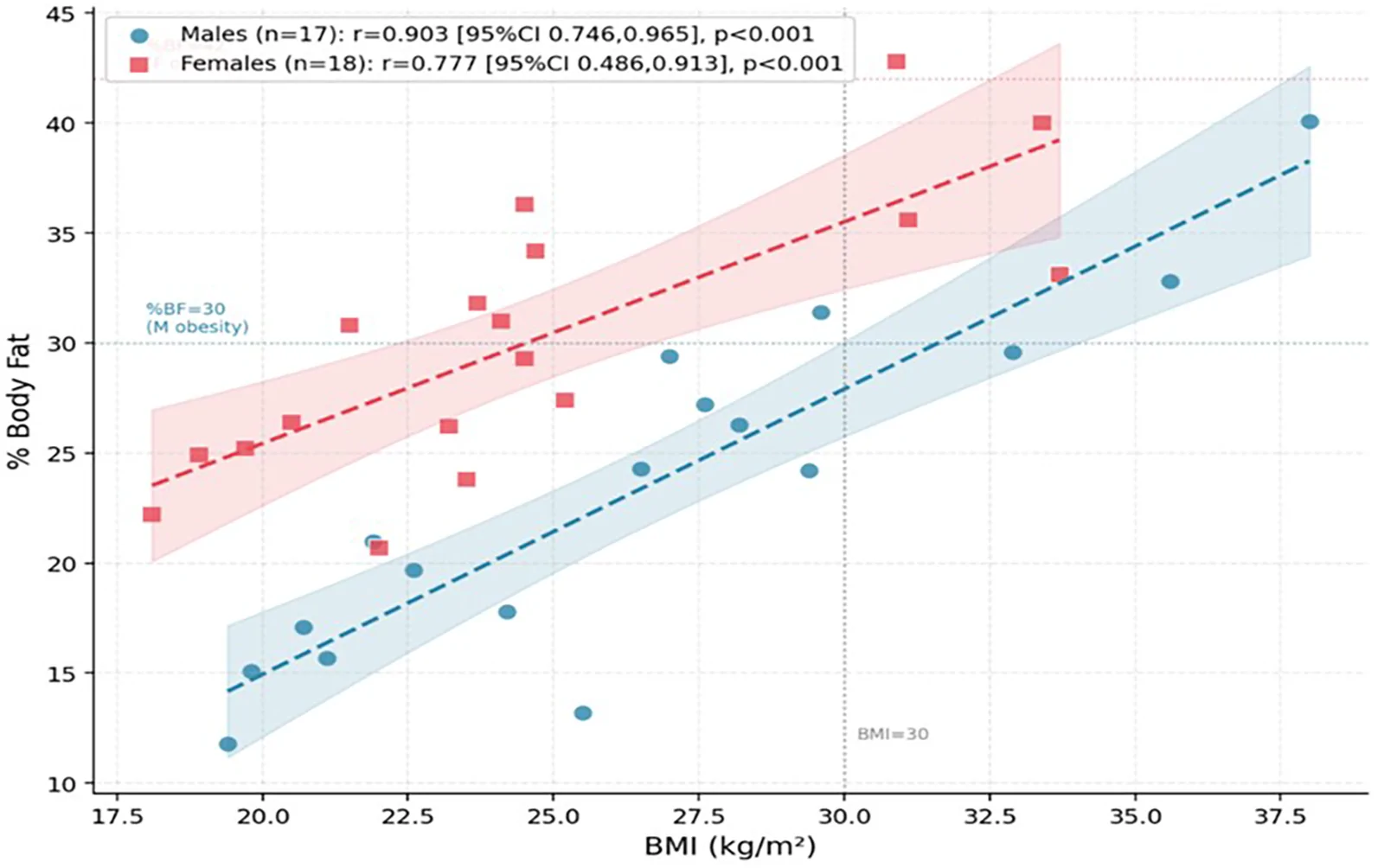
Pearson correlation: BMI vs. %BF by Sex (Shaded = 95% confidence band; reference lines = obesity thresholds).

BMI classified 15/35 patients [42.9% (95% CI: 28.0–59.1%)] as overweight/obese (8 overweight, 7 obese), compared to 10/35 [28.6% (95% CI: 16.3–45.1%)] by %BF (6 overweight, 4 obese) (Figure 1). The 14.3 percentage-point difference was not statistically significant [McNemar *p* = 0.125; *b* = 6 (BMI overclassifies), *c* = 1 (%BF overclassifies)]. On 3-level concordance, 25/35 (71.4%) received identical category assignments; 8 (22.9%) were assigned a higher BMI than %BF category, and 2 (5.7%) the reverse. Males had higher overweight/obesity prevalence by both methods than females, though not significantly so (BMI: 58.8% vs. 27.8%, *p* = 0.130; %BF: 41.2% vs. 16.7%, *p* = 0.219). Weighted kappa indicated moderate-to-substantial agreement [*κ* = 0.604 (95% CI: 0.303–0.834)]; higher in males (*κ* = 0.746) than females (*κ* = 0.421, CI crosses zero reflecting small n). Bland–Altman analysis showed no mean bias (difference = 0.000) and no proportional bias (slope = 0.000, *p* = 1.00), but wide limits of agreement (±1.601 SD), confirming the methods are not interchangeable at the individual level. Overall BMI–%BF correlation was *r* = 0.666 [95% CI: 0.428–0.818], *p* < 0.001. Sex-stratified correlations were *r* = 0.903 in males [0.746–0.965] and *r* = 0.777 in females [0.486–0.913] (both *p* < 0.001) (Figure 2). In patients ≤20 years (*n* = 24), BMI-SDS correlated more strongly with %BF than raw BMI (*r* = 0.723 overall; *r* = 0.856 males, *r* = 0.880 females).

## 4 Discussion

For a good number of years investigators have recognized BMI as being a simple, yet not ideal method of appreciating obesity, hence the continuous search for a more clinically relevant method based on other body characteristics. Our study, while not quite reaching statistical significance likely due to its small sample size, suggests that BMI might be overestimating obesity in this population. According to our results, the BMI method classified 42.9% of participants as overweight or obese compared to 25.7% using %BF method. Although not reaching statistical significance, potentially significant differences may have emerged with a larger sample.

The cut-offs for defining obesity proposed by Potter et al. in adults will need to be validated in AYAs with KTs as well. The definition of being overweight/suffering from obesity proposed by Potter et al. was based on a very large population and making a simple assumption (17). Basically, patients with metabolic syndrome were identified according to the definition of having at least 3 of the following different markers: increased waist circumference (men: >102 cm/>40 inches; women: > 88 cm/>35 inches), elevated triglycerides(>150 mg/dL/1.7 mmol/L), low HDL (men: <40 mg/dL; women: <50 mg/dL), hypertension (BP: systolic >130 mmHg and/or diastolic >85 mmHg) and elevated fasting glucose levels (≥110 mg/dL, or on medications for elevated glucose). Potter et al. derived sex-specific %BF thresholds by identifying %BF values corresponding to the 5% and 35% metabolic syndrome prevalence rates observed at BMI-defined overweight and obesity cutoffs in a large NHANES adult cohort (17). In our cohort, metabolic syndrome prevalence was 25.7% overall (22.2% overweight, 44.4% obese by %BF), differing from these reference rates, reflecting the unique cardiometabolic burden of KT recipients. Validation of these %BF cutoffs in a large AYA KT population, ideally multicenter, is needed.

If anything, the %BF method of defining being overweight or suffering from obesity is at least an alternative method that takes into account sex differences, as opposed to BMI method which does not differentiate between sexes. %BF showed significant sex differences: males 23.3 ± 7.9%; females 30.1 ± 6.1% (*P* < 0.05) (Table 2). This difference reflects normal male/female body composition differences previously described in the literature, but never in AYAs with KTs. Overall, men showed a stronger correlation (*r* = 0.903) than women (*r* = 0.777) between the %BF and the BMI methods (Figure 2). In addition, not all the women who were classified as overweight by %BF method were put in the same category by the BMI method, as some were considered normal weight (Figure 1C). This emphasizes the need for us to find a different method for defining overweight/obesity than BMI, a method that is sex-specific.

Assuming that our results can apply to pre-transplant candidates, this might open more doors for adolescents who are seeking a KT and are currently denied the opportunity due to their BMI. One of the criteria that selection committees generally look at before activating patients prior to KT is a BMI within a certain range (3). While the BMI cut-off criteria vary to some degree between transplant centers, a survey by the American Society of Transplantation reported that 66 out of 67 centers used BMI in selection criteria; the range of 35–45 Kg/m^2^ as the upper limit for acceptance was used (22). Defining obesity based on %BF instead might provide access to KT to more patients who otherwise would have been classified as obese based on the BMI definition.

A major limitation of our study was the modified definition of metabolic syndrome that we used. Considering the mean age (19.5 years) of our patients and the fact that 26 out of 35 total enrolled patients (74.3%) were at or above the age of 18 (Table 1) we have used the Pediatric guidelines for definition of the metabolic syndrome for patients up to age 18 (20) and the Adult Treatment Panel III (ATPIII) guidelines to define the metabolic syndrome for patients over 18 (21). According to the pediatric criteria, metabolic Syndrome is technically defined as the presence of being overweight or obese plus at least 2 of the 4 additional cardiovascular risks: systolic and/or diastolic BP ≥ 90th centile for age, sex and height or ≥130/80 mmHg, whichever is lower, or on anti-hypertensive medication; fasting triglycerides ≥100 mg/dL if age <10 years, or ≥130 mg/dL if age ≥10 years; fasting HDL <40 mg/dL; fasting serum glucose ≥100 mg/dL or known type 2 diabetes mellitus (20). According to the criteria for adults, metabolic syndrome is defined as the presence of any 3 or more of the following 5 clinical criteria: abdominal obesity (waist circumference) > 102 cm in men and >88 cm in women; elevated BP ≥130 mmHg systolic or ≥85 mmHg diastolic, or on antihypertensive medication); elevated triglycerides ≥150 mg/dL or on medication for high triglycerides; reduced HDL cholesterol <40 mg/dL in men and <50 mg/dL in women or on medication; elevated fasting glucose: ≥110 mg/dL, or previously diagnosed with type 2 diabetes) (21). However, for practical reasons, we chose slightly different components to define metabolic syndrome, namely 3 out of the following 5 criteria: obesity defined according to age (no abdominal circumference taken into account), elevated blood pressure according to age, elevated non-fasting triglycerides according to age, low HDL according to age and abnormal HbA1c (as a substitute for fasting glucose). This is because abdominal obesity and fasting glucose pose technical difficulties in our day-to-day practice, making consistent data collection nearly impossible. Waist circumference was not collected due to patient refusal, which may lead to a modest underestimation of metabolic syndrome prevalence. Working with a population that includes AYA females, getting patients to accept having their waist circumference measured was a very delicate issue, leading to a very high rate of refusals, a problem that was previously described in the literature (23). Future studies should implement standardized waist measurement protocols to address this gap. Fasting glucose should ideally have been used as per guidelines, but considering the patients’ age group and the varied time intervals at which they present in our Transition Clinic, it becomes practically difficult to track their fasting glucose. We therefore utilized the HbA1c as the closest substitute, which provides a long-term picture of the glucose metabolization in the body. In addition, it is to be noted that triglycerides levels were not obtained after fasting for the same reason described above. We do not think this would have had a significant impact on the results, as it was previously well-established that typically triglyceride levels increase by 17.7–35.4 mg/dL two to six hours after a meal (24–26). In fact, certain organizations have been recently advocating for rather checking non-fasting triglyceride levels (27, 28).

In addition, the sample size is small. *Post-hoc* power analysis revealed 34.2% power with *N* = 35 at *α* = 0.05, supporting characterization as a hypothesis-generating pilot study. Approximately 75 patients would be required for 80% power and ∼85 for 90% power. Given the reality of only having limited numbers of KT patients at a pediatric center, a multicenter study will definitely be needed to confirm our hypothesis (21, 23).

In conclusion, measuring %BF using a body composition scale in a clinical setting was feasible and required minimal equipment, staffing and time, working very well with the clinic flow. In our population of AYA KT recipients, none of the patients were underweight. Overweight or obesity were present in roughly one third of the patients, being more common in males. Abnormal hemoglobin A1c and lipids were very common, and so was hypertension and the full metabolic syndrome. Overall, the BMI-based method appeared to overestimate being overweight or obese, although not reaching statistical significance. Potentially significant differences may have emerged with a larger sample. %BF was higher in females and the correlation between the %BF and the BMI definition of being overweight/obese was stronger in men. Further studies are needed to confirm if %BF is more accurate in determining obesity compared to BMI in this population. This would be particularly important when patients are referred for transplantation, as for most centers, BMI above a certain cut-off is considered a contraindication. Defining obesity based on %BF instead might provide access to transplantation to more patients who otherwise would have been classified as obese based on the BMI definition.

## Data Availability

The original contributions presented in the study are included in the article/Supplementary Material, further inquiries can be directed to the corresponding author.
